# Dual-energy X-ray absorptiometry scans accurately predict differing body fat content in live sheep

**DOI:** 10.1186/s40104-018-0295-4

**Published:** 2018-11-12

**Authors:** David W Miller, Ellen J Bennett, Joanne L Harrison, Fiona Anderson, Clare L Adam

**Affiliations:** 1 School of Veterinary and Life Sciences, Murdoch University, 90 South St, Murdoch, WA 6150 Australia; 2 Scotland’s Rural College (SRUC), Peter Wilson Building, Kings Buildings, West Mains Road, Edinburgh, EH9 3JG UK; 3 Present address: The Faculty of Health, University of Tasmania, 17 Liverpool St, Hobart, TAS 7000 Australia; 4 School of Biological Sciences, University of Aberdeen, Tillydrone Ave, Aberdeen, AB24 3UU UK; 5 Rowett Institute of Nutrition and Health, University of Aberdeen, Foresterhill, Aberdeen, AB25 2ZD UK

**Keywords:** BCS, DEXA, Fat, Leptin, Longitudinal, Sheep

## Abstract

**Background:**

There is considerable interest in implementing mobile scanning technology for on-farm body composition analysis on live animals. These experiments evaluated the use of dual energy X-ray absorptiometry (DXA) as an accurate method of total body fat measurement in live sheep.

**Results:**

In Exp. 1, visceral and whole body fat analysis was undertaken in sheep with body condition scores (BCS) in the range 2 to 3.25 (scale 1: thin to 5: fat). The relationship of BCS was moderately correlated with visceral fat depot mass (*r* = 0.59, *P* < 0.01, *n* = 24) and whole body fat (*r* = 0.70, *P* < 0.001, *n* = 24). In Exp. 2, sheep with BCS in the range 2.25 to 3.75 were blood sampled to analyse circulating leptin concentrations, and were DXA scanned immediately post mortem for total body fat. Plasma leptin concentrations had low correlations with BCS (*r* = 0.50, *P* < 0.05, *n* = 17) and DXA body fat (*r* = 0.42, *P* < 0.05, *n* = 17), and no correlation with chemical body fat (*r* = 0.17, *P* > 0.05, *n* = 9). There was a moderate correlation between DXA body fat and BCS (*r* = 0.70, *P* < 0.01, *n* = 17), and DXA body fat was highly correlated with chemical body fat (*r* = 0.81, *P* < 0.001, *n* = 9). In Exp. 3, a series of five DXA scans, at 8-week intervals, was performed on growing sheep over a 32-week period. The average BCS ranged from 2.39 ± 0.07 (S.E.M.) to 3.05 ± 0.11 and the DXA body fat (%) ranged from 16.8 ± 0.8 to 24.2 ± 1.2. There was a moderate correlation between DXA body fat and BCS over the 32 weeks (*r* = 0.61, *P* < 0.001, *n* = 24).

**Conclusions:**

Overall, these experiments indicated that there was good agreement between BCS, DXA and chemical analysis for measuring total body fat in sheep, and that DXA scanning is a valid method for longitudinal measurement of total body fat in live sheep.

## Background

The definitive measure of total body fat content in sheep is by chemical fat analysis of the whole body, post mortem. However, an accurate objective measurement of the body fat content of live sheep is desirable in a number of agricultural and experimental situations, for example as a reproductive and growth management tool [1], and to predict total carcass lean/fat content. Currently, body condition scoring (BCS) is the accepted method of estimating body fatness in live sheep. This provides an adiposity score from assessment by palpation of the prominence and degree of cover of the spinous and transverse processes of the anterior lumbar vertebrae [2, 3]. Although accurate and repeatable with an experienced operator, BCS is inevitably subjective. Another, more objective, indicator of fatness in live sheep is the determination of the concentration of leptin, a hormone that is produced by adipose tissue [4]. The circulating concentration of leptin has been indicated to be a moderate indicator of back-fat thickness in sheep [5], although it is also affected by other factors such as food intake [6], and requires the invasive and time-consuming process of obtaining and analysing blood samples. More objective methods using non-invasive scanning technologies could be developed for research or ‘on-farm’ use.

Subcutaneous backfat depth measured by ultrasound scanning is also an ‘on-farm’ method of predicting carcass lean/fat content [7]. However, ultrasound has some problems and limitations for utility in sheep such as low accuracy and precision of measurement because of the small size and limited variation in subcutaneous fat thickness and longissimus muscle area in sheep, and the mobility of the soft subcutaneous fat layer, with wool an added complicating factor [8]. But for live sheep, the high cost/limited access, to date, of some of the more precise methods for evaluation of body composition, such as X-ray computer tomography (CT) or dual energy X-ray absorptiometry (DXA), has meant that ultrasound has been preferentially used in selection indexes to improve body composition [9, 10], despite its relatively low precision.

Dual-energy X-ray absorptiometry (DXA) scanning was originally developed to measure bone density in humans, but it went on to be used for measuring body composition [11]. The results are based on the differential attenuation of low and high energy X-rays by bone, fat and other soft tissues. DXA scanners have also been used in agricultural species to assess the composition of pig and sheep carcasses [12–17]. However, there are very limited data showing calibration of the technique, comparing DXA scan results with chemical carcass fat analysis, or data comparing DXA scanning with BCS or with whole body fat [14, 17–20], and no information on conducting repeated measurements over time for live sheep. Here we compare DXA scanning to BCS, circulating leptin concentrations and post mortem fat analysis to estimate total body fat content. The objectives of the three experiments were to: 1) confirm the relationship between BCS measurements in sheep and whole-body fat content measured by chemical analysis; 2) extend the findings of Exp. 1 with additional blood leptin measurements and DXA scan data; and 3) determined the longitudinal relationship between DXA fat measurements and BCS in live growing sheep.

## Methods

These experiments were conducted under the authority of the UK Animals (Scientific Procedures) Act of 1986 (PPL 2854: 19b # 4, 5, 6) and received prior approval from the local Ethical Review Committee. All sheep were Suffolk x Greyface castrated males.

### Exp. 1: Comparison of BCS to post mortem body fat analysis

Sheep (*n* = 24) aged 12 months were used that had been kept for 6 weeks on either an ad libitum (*n* = 12) complete diet (10 MJ ME/kg DM, comprising 50% coarsely milled hay, 30% rolled barley, 9% soyabean meal, 10% molasses and added mineral and vitamin supplements) to promote growth, or a restricted complete diet (*n* = 12) calculated to provide 50% of that required to maintain current body mass and adiposity. Final body mass ranged from 46 to 66 kg and BCS ranged from 2 to 3.25 (scale 1: thin to 5: fat; [2, 3]; NB: same experienced person conducted all BCS). They were killed by lethal intravenous dose of sodium pentobarbitone (Euthesate, Willows Francis Veterinary, Crawley, Sussex, UK). The head, internal organs, pelt and lower limbs were removed; visceral fat (omental and mesenteric) was stripped from the gastrointestinal tract. Mass was recorded for this adipose tissue depot and it was placed back with the carcass, which then underwent chemical composition analysis.

### Exp. 2: Comparison of BCS, leptin, chemical body fat analysis and DXA

Sheep (*n* = 17) aged 14 months were used that had been kept for 12 weeks on either ad libitum or restricted (50% maintenance) diet. Final body mass was in the range 63 to 94 kg and BCS 2.25 to 3.75, and they were killed as above. The intact body, including pelt and internal organs, was immediately positioned in sternal recumbency on the bed of the DXA scanner and a whole body scan was performed within 5 min of euthanasia. Blood samples were also taken on the day before each DXA scanning via jugular venepuncture, and analysed for leptin concentration by homologous radioimmunoassay [21] in a single assay run with sensitivity 0.05 ng/mL and an intra-assay CV of 7.1%. The length of wool on the pelt was approximately 40 mm (with a typical fibre diameter between 25 to 35 μm). After DXA scanning, the carcasses from a subset of the sheep (*n* = 9), selected to represent the range of BCS, underwent chemical composition analysis.

### Carcass analysis

The carcasses (without pelt, head and lower limbs, but including digesta-free viscera and visceral fat - in order to match more closely the whole body in vivo) were individually homogenised in a Wolfking mincer (Slagelse, Denmark) [22]. After mixing, three samples of each homogenate were freeze-dried, ground in a laboratory mincer, and subsampled for chemical composition analyses. The fat content was determined by solvent extraction with petroleum ether (Soxtec 2050, Tecator, Höganäs, Sweden). The residual moisture content of the carcass mince remaining after freeze-drying was obtained by drying to constant mass at 100 °C. The fat content was determined by the chloroform-methanol technique [23].

### Exp. 3: Comparison of BCS and DEXA in live growing sheep

Sheep (*n* = 24) aged 6 months and initial body mass 44 ± 2.9 kg (S.D.) and BCS 2.4 ± 0.34 (S.D.) were individually housed and offered the ad libitum complete diet to promote growth. Body weight and BCS were recorded and DXA scanning was carried out at 0, 8, 16, 24 and 32 weeks. The sheep were shorn about 3 weeks prior commencement of the experiment, so the length of wool was approximately 15 mm at week 0 and 75 mm by week 32. DXA scanning was conducted during the morning; water was not withheld beforehand, but the provision of fresh food was delayed until after the scan. Each sheep was anaesthetised with 5% to 6% halothane (Halothane BP, Concord Pharmaceuticals Ltd., Essex, UK), placed in sternal recumbency on the scanner bed and maintained on ~ 2% halothane via the mask for the duration of the scan (15 to 20 min).

### DXA scanner

The scanner used was a Norland XR-26, Mark II, high-speed pencil beam scanner equipped with dynamic filtration (Norland Corporation, Fort Atkinson, WI, USA). The integral software analysed the whole body DXA scan data to provide estimates of total fat mass and total fat percentage. The CV of 3.6% for the DXA whole body fat percentage estimate, obtained by conducting 2 repeat scans on the same day in each of 9 individuals with DXA total body fat readings of 16% to 30%, was close to that obtained for humans using the same type of Norland scanner (2.6%; [24]).

### Statistical analysis

Pearson product-moment correlation analyses were used to explore the relationships between measured variables (Minitab). Residuals were checked for constant variance and normality of distribution. For prediction analysis, chemical fat was analysed using a general linear model (SAS Version 9.1, SAS Institute, Cary, NC, USA) with covariates included one at a time for DXA fat, BCS and leptin. The prediction of visceral fat was analysed using a general linear model with BCS included as covariate. To analyse the change in BCS over time a linear mixed effects model was used with DXA fat included as a covariate, time as a fixed effect and animal identification included as a random term to account for repeated measures. Repeated measures analysis of variance (General Linear Model; Minitab 16, Pennsylvania, USA) was carried out on the BCS and liveweight data, with time as the fixed effect and animal as the random effect. Post-hoc Fisher’s protected least significant difference analysis was used to test for specific differences between treatments at each time point.

## Results

### Exp. 1: Comparison of BCS to post mortem body fat analysis

Prior to post mortem fat analysis, the BCS of the sheep ranged from 2 to 3.25. Dissected visceral adipose depot mass ranged from 110 to 1,055 g (fresh mass). Total body fat by chemical analysis (% body mass) ranged from 12.6% to 28.1%. The linear relationship of visceral fat depot mass was highly correlated with whole body fat (%) determined by chemical analysis (*r* = 0.86, *P* < 0.001).

**Fig. 1:**
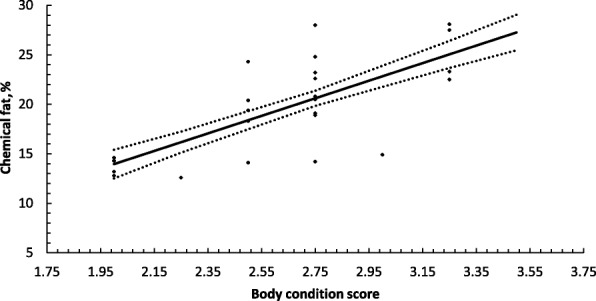
Prediction of chemical fat (%) in sheep using body condition scoring. Solid line represents least squared means ± S.E. (dotted lines) with marker indicating residuals from this relationship, *R*^2^ = 0.49, RMSE 3.63

**Fig. 2:**
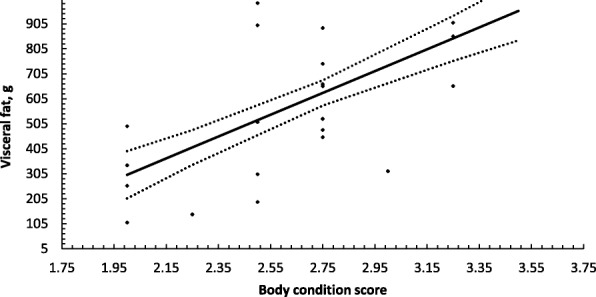
Prediction of visceral fat (g) using body condition scoring. Solid line represents least squared means ± S.E (dotted line) with marker indicating residuals from this relationship, *R*^2^ = 0.359 RMSE 238.7

There was moderate ability of BCS to predict chemical fat (*P* < 0.01, *R*^2^ = 0.49, RMSE 3.63), with this relationship shown in Fig. 1. The relationship between BCS and visceral fat was not as strong (*R*^2^ = 0.36, RMSE 238.7), with this relationship shown in Fig. 2.

### Exp. 2: Comparison of BCS, leptin, chemical body fat analysis and DXA

Prior to post mortem fat analysis, the BCS of the sheep ranged from 2.25 to 3.75 (*n* = 17), and circulating plasma leptin concentrations ranged from 1.5 to 12.7 ng/mL (*n* = 17). DXA total body fat percentage values ranged from 19.1% to 32.9% (*n* = 17). There was a moderate correlation between DXA body fat and BCS (*r* = 0.70, *P* < 0.01), while plasma leptin concentrations had low correlations with BCS (*r* = 0.50, *P* < 0.05) and DXA body fat (*r* = 0.42, *P* < 0.05). For the subset (*n* = 9) of sheep selected for chemical body fat analysis, the BCS ranged from 2.25 to 3.75, and circulating plasma leptin concentrations ranged from 1.5 to 11.6 ng/mL. DXA total body fat percentage values in the subset of sheep ranged from 19.1% to 30.3%, and total body fat percentage by chemical analysis ranged from 19.8% to 31.7%.

**Fig. 3:**
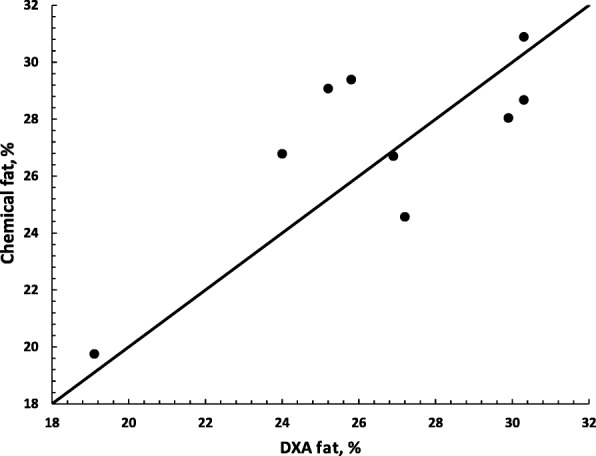
Relationship between DXA predicted fat (%) and actual chemical fat (%). DXA fat = dual-energy absorptiometry-determined body fat (%). Line represents perfect relationship with solid dots showing the residuals from this relationship, *R*^2^ = 0.58 RMSE 2.29

The chemical body fat was predicted by DXA with moderate precision (*P* < 0.05, *R*^2^ = 0.58, RMSE 2.29) with this relationship shown in Fig. 3. In this experiment the prediction of chemical fat using BSC was less precise than that of DXA (*P* = 0.07, *R*^*2*^ = 0.38, RMSE 2.79). The was poor ability of plasma leptin to predict chemical fat (*P* > 0.05) There was a moderate correlation between BCS and DXA body fat (*r* = 0.74, *P* < 0.001), and a low correlation between plasma leptin concentration and DXA body fat (*r* = 0.44, *P* < 0.05).

### Exp. 3: Comparison of BCS and DEXA in live growing sheep

**Fig. 4:**
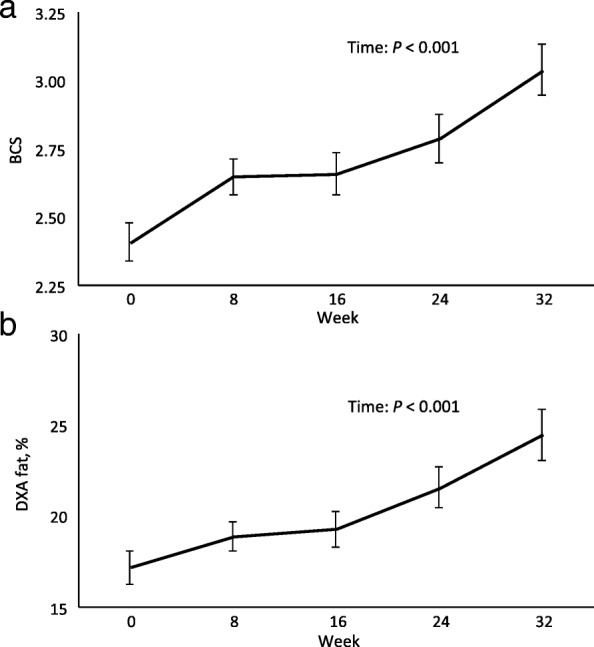
Longitudinal (**a**) body condition score and (**b**) dual-energy absorptiometry-determined body fat in growing sheep. BCS = body condition score. DXA fat = dual-energy absorptiometry-determined body fat (%). Six-month through to 14-month old castrated male sheep. Measurements taken every 8 weeks over 32 weeks (*n* = 24, Exp. 3). Values are means ± S.E.M. The significant effect of time is presented as: Time: *P* < 0.001

There was a significant overall effect of time on BCS and DXA total body fat percentage (both *P* < 0.001; Fig. 4), with average BCS increasing from 2.39 ± 0.07 (S.E.M.) to 3.05 ± 0.11 (range: 2 to 3.25), and the average DXA body fat (%) increasing from 16.8 ± 0.8 to 24.2 ± 1.2 (range: 7.7 to 28.6) over the 32 weeks.

At week 0 there was a high correlation between DXA body fat and BCS (*r* = 0.72, *P* < 0.001, *n* = 24, Table 1), low correlation at week 8 (*r* = 0.48, *P* < 0.05), moderate correlation at week 16 (*r* = 0.62, *P* < 0.01), moderate correlation at week 24 (*r* = 0.58, *P* < 0.01), and a moderate correlation at week 32 (*r* = 0.64, *P* < 0.01). When the data from all time-points were analysed together there was a moderate correlation between DXA body fat and BCS over the 32 weeks (*r* = 0.61, *P* < 0.001).

**Table 1:** The longitudinal association between body condition score and dual-energy X-ray absorptiometry-determined total body fat performed on live growing sheep

Time	BCS vs DXA fat
Week 0	0.72***
Week 8	0.48*
Week 16	0.62**
Week 24	0.58**
Week 32	0.64***
Overall (weeks 0–32)	0.61***

BCS = body condition score. DXA fat = dual-energy X-ray absorptiometry-determined total body fat (%). Six-month through to 14-month old castrated male sheep. Measurements taken every 8 weeks over 32 weeks (*n* = 24, Exp. 3). The associations are represented as correlation coefficients (*r*). Asterisks indicate statistical significance at: *P < 0.05, **P < 0.01, ***P < 0.001

## Discussion

These experiments have demonstrated good agreement between different measures and estimates of body fat in sheep across a range of nutritional states and have validated the use of DXA scanning to estimate total body fat content in live, growing sheep.

The first experiment confirmed a good positive correlation, as also reported by Russel et al. [2], between BCS measurements in sheep and whole-body fat content measured by chemical analysis. Although subjective, BCS remains a valuable and practical means of in vivo body fat assessment in sheep. However, there is obvious inaccuracy in the method, especially in the mid-range of the BCS measurement scale. This has long been known to be an inherent weakness in the BCS method [25]. In the present study, the majority of the error occurred around the middle of the BCS measurement range. This BCS mid-range is important for improving production outcomes, e.g. increasing the condition of Merino ewes at joining from 2.5 to 3 can increase the number of lambs born by 15% [26]. Therefore, an increase in precision and accuracy of adiposity measurement by using a more objective measure than BCS may have significant industry benefits.

The second experiment extended the findings of Exp. 1, in a different cohort of sheep, with additional blood leptin measurement and DXA scan data for comparison. DXA total body fat estimates were closely correlated with BCS and highly correlated with whole body chemical fat analysis, thereby supporting its use as an objective measure of total body fat content. DXA scanners have been used previously to assess sheep carcasses [13, 14, 16, 18, 19], showing it to be a reliable technique to analyse body composition. Indeed, next generation DXA scanners are now being used in Australian abattoirs, scanning at speeds equalling chain speeds of 16.6 cm/s, to generate accurate coordinates for robotic carcass cutting and boning [27]. Pearce et al. [17] compared DXA scanning of live sheep with carcass fat analysis conducted on the animals that were slaughtered 10 d after scanning. In their study, they found a moderate level of prediction of carcass fat (i.e. *R*^2^ = 0.7, RMSD 0.71 kg) from live DXA scanning.

Comparisons to circulating leptin concentrations were also obtained in Exp. 2 and these indicated that there were poor correlations between BCS, DXA fat and leptin. A previous study indicated that the circulating concentration of leptin was a moderate indicator of back-fat thickness in sheep [5], and in Exp. 1 we found DXA to be highly correlated to visceral fat mass, which is known to be a determinant of plasma leptin levels in humans [28]. The difference in our study may be due to our sheep being in a dynamic rather than fixed state of adiposity. We have previously shown circulating levels to be poorly correlated to BCS in sheep on an increasing or decreasing nutritional plane [6]. Moreover, it is known that the central orexigenic pathways that homeostatically control circulating leptin concentrations are more sensitive to a changing nutritional status than to absolute nutritional status (body condition) [6].

Data from the sheep in Exp. 3 further expanded on the findings from Exps. 1 and 2, with significant positive correlations between BCS and DXA fat measurements. Importantly, Exp. 3 also demonstrated, for the first time, that the positive correlation between DXA fat measurements and BCS holds true longitudinally in vivo. Given the high correlation between DXA and chemically-determined whole body fat shown in Exp. 2, it can be postulated that DXA scanning could be a reliable (on-farm) method for measurement of longitudinal changes in total body fat content in live sheep.

## Conclusions

Overall, there was good agreement between BCS, DXA and chemical analysis for measuring total body fat content in sheep, and we have demonstrated that DXA scanning is a valid method for longitudinal measurement of total body fat content in live sheep. The application of DXA for estimating total body fat content, and carcass fat content, in live animals has obvious potential as a research and management tool. Moreover, with next generation DXA technologies that are currently being implemented in abattoirs in Australia with speeds of analysis greater than 10 bodies per minute [27], a mobile DXA (on-farm) measurement for live animal body fat content estimation and carcass lean/fat content prediction could be a real possibility. Future work would involve the use of a larger data set representing a wider range of sheep body condition scores, with comparisons made between body condition score, measured body fat and DEXA predicted body fat.

## Abbreviations

BCS: Body condition score
CT: Computed tomography
DXA: Dual-energy X-ray absorptiometry
RMSE: Root mean square error
